# Editorial: Clinical implementation of precision oncology data to direct individualized and immunotherapy-based treatment strategies

**DOI:** 10.3389/fimmu.2025.1631591

**Published:** 2025-06-13

**Authors:** Nicholas A. Young, Jenifer R. Prosperi, Aharon G. Freud, Nelson S. Yee, Emanuel F. Petricoin

**Affiliations:** ^1^ Division of Rheumatology and Immunology, Department of Internal Medicine, The Ohio State University Wexner Medical Center, Columbus, OH, United States; ^2^ More than Moore’s Precision Medicine Solutions (MtM), Columbus, OH, United States; ^3^ Private Health Management (PHM), Los Angeles, CA, United States; ^4^ Department of Biological Sciences, University of Notre Dame, Notre Dame, IN, United States; ^5^ Harper Cancer Research Institute, South Bend, IN, United States; ^6^ Department of Biochemistry and Molecular Biology, Indiana University School of Medicine, South Bend, IN, United States; ^7^ Department of Pathology, The Ohio State University Wexner Medical Center, Columbus, OH, United States; ^8^ Division of Hematology-Oncology, Department of Medicine, Pennsylvania State University College of Medicine, Hershey, PA, United States; ^9^ Next-Generation Therapies Program, Penn State Cancer Institute, Penn State Health Milton S. Hershey Medical Center, Hershey, PA, United States; ^10^ Ignite Proteomics Inc., Golden, CO, United States; ^11^ Center for Applied Proteomics and Molecular Medicine, George Mason University, Manassas, VA, United States

**Keywords:** precision oncology, immunotherapy, individualized patient treatment, tumor-agnostic clinical/molecular testing, molecular profiling, next-generation sequencing (NGS) (DNAseq/RNAseq), pan-omics, drug sensitivity testing

## Abstract

Graphical summary.

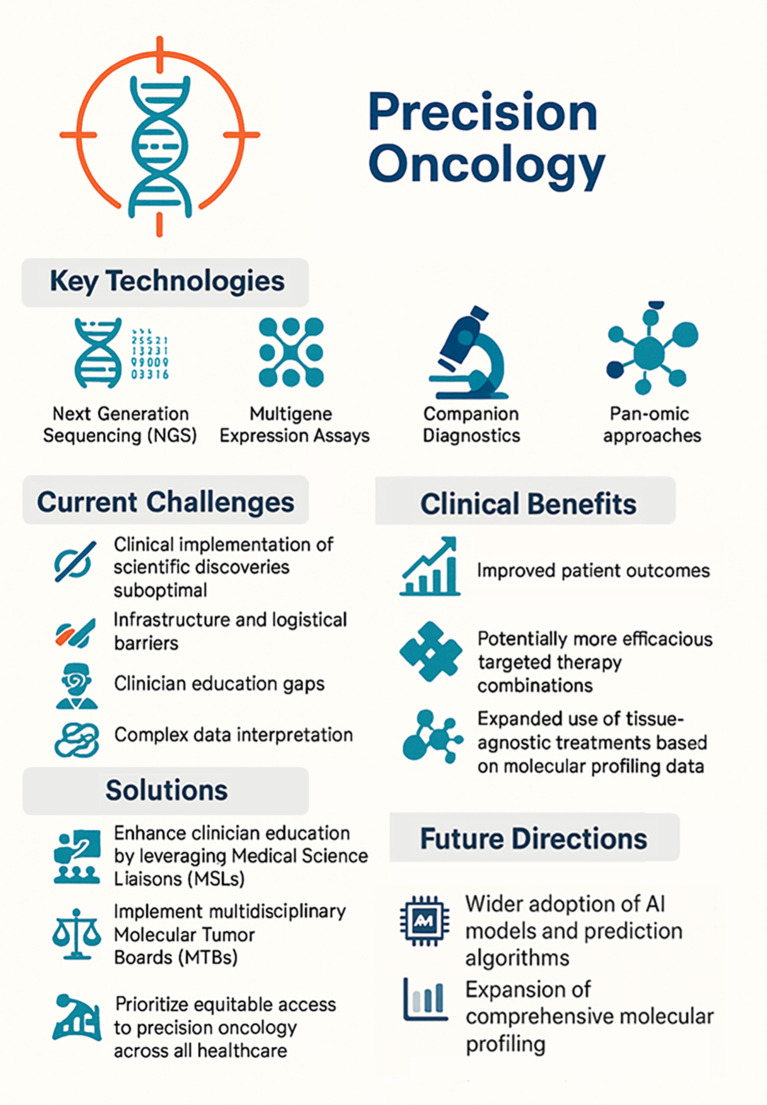

## 1 Introduction

Precision oncology has become a core component of cancer care and can be broadly defined as the use of molecular profiling data from clinically approved testing to inform therapeutic decision making or to stratify patients based on predicted treatment responses or toxicities. Technologies such as next-generation sequencing (NGS), multigene expression assays, and companion diagnostics are now routinely used to identify actionable mutations and optimize therapy selection for individual cancer patients (1). Accordingly, clinical practice guidelines (e.g., National Comprehensive Cancer Network) recommend comprehensive biomarker testing for nearly all patients with advanced or metastatic cancer to help identify the most effective treatments (2). However, despite recent scientific advancements and guideline recommendations, clinical implementation of emerging molecular testing strategies has been suboptimal. While there are obstacles to establishing precision oncology workflows even in large healthcare networks (3), community oncology settings (where over 80% of cancer patients receive care in the USA) face more significant barriers to adoption. These include delays in tissue access, limited tissue availability, and lack of the infrastructure that is required for comprehensive molecular testing, sample pre-processing/shipment or storage, and ordering (4). Consequently, while individualized treatment strategies based on precision oncology analysis that considers molecular profiling and other clinical testing data are technically, logistically, and financially achievable, this approach is not comprehensively implemented currently.

In addition to NGS-based methods to profile both somatic and germline alterations, the past decade has also seen the development and clinical approval of multiple technologies and pan-omic approaches to interrogate disease. These data provide robust information to identify clinically actionable targets and potentially predict treatment efficacy or sensitivity/resistance. The first tissue-agnostic drug in oncology (pembrolizumab) was approved by the United States Food and Drug Administration (FDA) in 2017 for the treatment of microsatellite instability (MSI)-High or mismatch repair deficient (dMMR) solid tumors. Since then, the field of precision oncology continues to grow as more drugs are approved based on molecular-specific testing independent from tissue of origin or disease indication/diagnosis (5). When patients are matched to the optimal targeted treatment, outcomes often improve compared to traditional chemotherapy or standard of care strategies. In a prospective study of treatment-naïve and advanced solid tumors, patients treated based on molecular profiling matching and Molecular Tumor Board (MTB) recommendations had higher disease control rates, longer progression-free survival, and improved overall survival than those treated with standard of care therapy (6). The conclusions of this trial were that administering personalized treatment earlier in the disease course may improve outcomes and targeted therapy combinations are more effective than mono-therapeutic approaches.

Since FDA approvals of cancer therapeutics will be more commonly tumor-agnostic moving forward, clinicians will require a better understanding of commercially available clinical tests to be confident in using the results to guide treatment decisions for their patients. However, the interpretation of data from comprehensive molecular testing and prioritization of testing modalities can be overwhelming, especially in a community oncology setting. Thus, clinician education and decision support are essential to bridge the gap between research and practice. Medical Science Liaisons (MSLs) are clinical scientists with expertise in molecular profiling who can educate physicians on emerging molecular testing and targeted therapeutics. Furthermore, multidisciplinary MTBs where oncologists, geneticists, pathologists, and other research specialists review complex cases to interpret genomic findings and discuss emerging therapeutic options can also be valuable for clinician education/exposure. Consequently, more comprehensive education initiatives, including MSLs and MTBs, are needed so that every oncologist is equipped with the knowledge and confidence to practice precision oncology. To begin to achieve this overarching goal, the objective of this Research Topic is to provide high-quality clinical evidence and summarize emerging molecular tests to help guide clinical adoption; publication of this Research Topic collection may drive efficient translation of scientific discoveries into clinical care and ultimately improve patient outcomes by facilitating effective implementation of precision oncology-based treatment strategies.

## 2 Research Topic summary

Developing precision medicine-guided immunotherapeutic strategies can improve patient outcomes by tailoring treatment to individual tumor biology and molecular profiling data. This approach was summarized in a mini-review by Chhabra that outlined the transformative impact of integrating molecular profiling, advanced diagnostics, and artificial intelligence (AI) into precision oncology practice, particularly in the context of immunotherapies. The additional works within this Research Topic include biomarker analyses, predictive modeling, case insights, and meta-analyses that support precision oncology diagnostics and personalized therapeutic strategies.

Several studies analyzed blood-based and tissue biomarkers to identify patients most likely to benefit from immunotherapies or combination regimens, thereby maximizing efficacy and minimizing toxicity. Li et al. demonstrated that peripheral blood biomarkers can predict prognosis and immune-related adverse events (irAEs) in patients with stage IV driver gene-negative lung adenocarcinoma treated with immune checkpoint inhibitors (ICIs). Huang et al. identified a glycolysis-related gene signature that stratified breast cancer patients by prognosis and immunotherapy responsiveness. Tang et al. demonstrated that the Systemic Inflammation Response Index (SIRI), a blood-based biomarker reflecting immune and inflammatory status, can serve as a reliable predictor of survival in advanced non-small cell lung cancer (NSCLC) patients undergoing PD-1 inhibitor immunotherapy. Guo et al. developed and validated a survival prediction model for patients with advanced NSCLC by integrating clinical, molecular, and immunologic biomarkers to enable clinicians to accurately tailor treatment decisions based on molecular and immune profiles. Wallen et al. found that younger patients had relatively low tumor mutational burden (TMB) and reduced expression of immune-related genes across many solid tumors, potentially limiting their response to ICIs. Importantly, their survival analyses revealed that younger males had worse outcomes on immunotherapy alone, but this was mitigated when chemotherapy was added, which suggests that age and sex should be considered in determination of combination strategies. Zhang et al. identified the fibrosis-4 index as a novel, accessible biomarker panel indicative of liver fibrosis for predicting poorer outcomes in patients with cholangiocarcinoma receiving immunotherapies, suggesting that liver health profoundly influences tumor response to immunotherapy.

Two studies published in this Research Topic evaluated adjunct data that may be used to enhance immunotherapy effectiveness for certain patient subpopulations. Wang et al. reviewed the interconnected roles of the nervous system, gut microbiota, and immune system in tumor development, highlighting the “nervous system–gut microbiota–immune system axis” as a promising target for cancer prevention and precision oncology-informed therapy. Ji et al. evaluated how hepatitis B virus (HBV) load impacts the effectiveness of ICIs in hepatocellular carcinoma (HCC) patients in a meta-analysis, providing key insights for improving individualized immunotherapeutic approaches.

The case reports of exceptional responders reinforce learning from outliers to expand immunotherapy treatment options for traditionally resistant cancers. Li et al. presented two rare instances of advanced pancreatic cancers with high TMB and/or PD-L1 expression that responded favorably to pembrolizumab. Both patients exhibited durable clinical responses following pembrolizumab treatment, despite their cancers traditionally being considered “immunologically cold” and non-responsive to immunotherapy. Yu et al. presented a case report with a remarkable complete response in a patient with metastatic cervical cancer treated with cadonilimab, a novel bispecific antibody targeting both PD-1 and CTLA-4. Despite the presence of PD-L1-negative metastases, the patient achieved a durable 10-month complete remission.

Other studies within this Research Topic collection used meta-analyses or retrospective datasets to stratify patient populations and develop methodologies to predict responsiveness to treatment. Yang et al. developed and validated a nomogram that integrates clinical and CT imaging features to predict the presence of disease spread through air spaces in patients with early-stage lung adenocarcinoma. Gan et al. evaluated research trends on combining immunotherapy and targeted agents in HCC and found that a dual-targeting approach involving ICIs and anti-angiogenic agents had particularly promising clinical efficacy, which correlates with emerging clinical data for liver cancer treatment. Yang et al. showed that a triple neoadjuvant combination with transarterial therapy, bevacizumab, and ICI significantly improved outcomes for patients with locally advanced HCC, indicating greater efficacy with an individualized approach integrating localized tumor targeting in combination with systemic immunomodulation. Felfi et al. established a novel tumor growth modeling approach to more accurately predict immunotherapy responses in NSCLC relative to the standard RECIST 1.1 criteria by integrating early tumor growth kinetics. Kang et al. described how antiangiogenic therapies (e.g., bevacizumab) enhance precision cancer treatment by modifying the tumor microenvironment (TME) to boost antitumor immunity and therapeutic efficacy. Wang et al. discovered that a history of thyroid carcinoma was associated with an increased risk of secondary malignancies, particularly bladder cancer, by integrating genome-wide association studies, RNA sequencing data, and immune infiltration analyses.

Collectively, these studies advance the field of precision oncology by identifying novel biomarkers, establishing predictive testing models, and evaluating novel therapeutic combinations that may facilitate better patient stratification and could be integrated into clinical care to improve patient outcomes. Thus, this Research Topic collection adds to the research demonstrating that integration of multi-dimensional patient and molecular profiling data is key to optimizing cancer prognostication and tailoring precision oncology treatment strategies.

## 3 Conclusions and future directions

Recent scientific advances in genomics, molecular diagnostics, and drug sensitivity testing have significantly outpaced clinical implementation, which has translated into suboptimal incorporation into routine care. Even in the most scientifically and medically advanced countries, comprehensive molecular testing is not widespread clinically and standardized molecular testing strategies to stratify patients with precision oncology-based treatments are still lacking. In the USA specifically, studies have shown that despite a majority of oncologists accepting that the field of precision oncology is an important and emerging part of the clinical environment with great growth potential, significantly less reported regularly using comprehensive genomic testing to guide treatment (7, 8). Challenges to clinical implementation of precision oncology include physician education and adoption, data analysis and interpretation, clinical trial access, and unknown costs associated with insurance coverage/reimbursement (9). As of 2025, the USA has a robust pipeline of targeted drugs and immunotherapies. Moreover, comprehensive genomic/molecular testing is more accessible than ever; by clinician education through MSLs and fostering structured collaborations through MTBs, the research-practice gap can be more effectively addressed. Importantly, access and equity should also be a focus so that precision oncology can be standard of care for all patients, including those in community hospitals and underserved areas (summarized in **Graphical Abstract**).

In conclusion, precision oncology is an emerging and under-practiced therapeutic approach that has demonstrated improved patient outcomes in limited studies by a more comprehensive understanding of cancer biology on a molecular level. Looking ahead, research discoveries will bring even more sophisticated precision medicine approaches to the clinic, including AI models and prediction algorithms, whole transcriptome RNA expression profiling, proteomic analyses, and functional precision oncology approaches (e.g., organoid drug sensitivity testing). The challenge moving forward will be to efficiently implement, scale, and optimize precision oncology utilization across all cancers and care settings.

## References

[1] Tsimberidou AM Fountzilas E Nikanjam M Kurzrock R. Review of precision cancer medicine: Evolution of the treatment paradigm. Cancer Treat Rev. (2020) 86:102019. doi: 10.1016/j.ctrv.2020.102019 32251926 PMC7272286

[2] Mateo J Steuten L Aftimos P André F Davies M Garralda E. Delivering precision oncology to patients with cancer. Nat Med. (2022) 28:658–65. doi: 10.1038/s41591-022-01717-2 35440717

[3] Dias-Santagata D Heist RS Bard AZ da Silva AFL Dagogo-Jack I Nardi V. Implementation and clinical adoption of precision oncology workflows across a healthcare network. Oncologist. (2022) 27:930–9. doi: 10.1093/oncolo/oyac134 PMC9632318 35852437

[4] Powell SF Dib EG Bleeker JS Keppen MD Mazurczak M Hack KM. Delivering precision oncology in a community cancer program: results from a prospective observational study. JCO Precis Oncol. (2018) 2:1–12. doi: 10.1200/PO.17.00220 35135120

[5] Thein KZ Myat YM Park BS Panigrahi K Kummar S. Target-driven tissue-agnostic drug approvals-A new path of drug development. Cancers. (2024) 16:2529. doi: 10.3390/cancers16142529 39061168 PMC11274498

[6] Sicklick JK Kato S Okamura R Patel H Nikanjam M Fanta PT. Molecular profiling of advanced Malignancies guides first-line N-of-1 treatments in the I-PREDICT treatment-naïve study. Genome Med. (2021) 13:155. doi: 10.1186/s13073-021-00969-w 34607609 PMC8491393

[7] Markham MJ Wachter K Agarwal N Bertagnolli MM Chang SM Dale W. Clinical cancer advances 2020: annual report on progress against cancer from the american society of clinical oncology. J Clin Oncol Off J Am Soc Clin Oncol. (2020) 38:1081. doi: 10.1200/JCO.19.03141 32013670

[8] de Moor JS Gray SW Mitchell SA Klabunde CN Freedman AN. Oncologist confidence in genomic testing and implications for using multimarker tumor panel tests in practice. JCO Precis Oncol. (2020) 4:620–31. doi: 10.1200/PO.19.00338 PMC7446310 32923869

[9] Schroll MM Agarwal A Foroughi O Kong E Perez O Pritchard D. Stakeholders perceptions of barriers to precision medicine adoption in the United States. J Pers Med. (2022) 12:1025. doi: 10.3390/jpm12071025 35887521 PMC9316935

